# Cross-Immunization Against Respiratory Coronaviruses May Protect Children From SARS-CoV2: More Than a Simple Hypothesis?

**DOI:** 10.3389/fped.2020.595539

**Published:** 2021-01-18

**Authors:** Pier Paolo Piccaluga, Giovanni Malerba, Mohsen Navari, Erica Diani, Ercole Concia, Davide Gibellini

**Affiliations:** ^1^Department of Experimental, Diagnostic, and Experimental Medicine, Bologna University School of Medicine, Bologna, Italy; ^2^Department of Pathology, School of Health, Jomo Kenyatta University of Agriculture and Technology, Nairobi, Kenya; ^3^Biomolecular strategies, genetics, cutting-edge therapies and neuroscience (SBGN), Euro-Mediterranean Institute of Science and Technology (IEMEST), Palermo, Italy; ^4^Department of Neurosciences, Biomedicine and Movement, Section of Biology and Genetics, Verona University, Verona, Italy; ^5^Department of Medical Biotechnology, School of Paramedical Sciences, Torbat Heydariyeh University of Medical Sciences, Torbat Heydariyeh, Iran; ^6^Research Center of Advanced Technologies in Medicine, Torbat Heydariyeh University of Medical Sciences, Torbat Heydariyeh, Iran; ^7^Bioinformatics Research Group, Mashhad University of Medical Sciences, Mashhad, Iran; ^8^Department of Diagnostic and Public Health, Unit of Microbiology, Verona University, Verona, Italy; ^9^Department of Diagnostics and Public Health, University of Verona, Verona, Italy

**Keywords:** SARS-CoV-2, coronaviruses, COVID-19, HCoV-OC43, cross-reactive immunity

## Abstract

In January 2020, a new coronavirus was identified as responsible for a pandemic acute respiratory syndrome. The virus demonstrated a high infectious capability and not-neglectable mortality in humans. However, similarly to previous SARS and MERS, the new disease COVID-19 caused by SARS-CoV-2 seemed to relatively spare children and younger adults. Some hypotheses have been proposed to explain the phenomenon, including lower ACE2 expression in children, cross-immunization from measles/rubella/mumps and BCG-vaccination, as well as the integrity of respiratory mucosa. Herein, we hypothesize that an additional mechanism might contribute to children's relative protection from SARS-CoV-2, the cross-immunization conferred by previous exposures to other common respiratory coronaviruses. To support our hypothesis, we show a statistically significant similarity in genomic and protein sequences, including epitopes for B- and T-cell immunity, of SARS-CoV-2 and the other beta coronaviruses. Since these coronaviruses are highly diffused across pediatric populations, cross-reactive immunity might reasonably induce an at least partial protection from SARS-CoV-2 in children.

## Introduction

Despite the significant improvements and the efforts made by governments and international organizations worldwide, the number of COVID-19 cases is still growing, and many issues are still unsolved, including definition of a standard care, development of effective immunization strategies, and proper epidemiological framework. Furthermore, the definition of COVID-19 as the cause of death varies across countries, depending on the role of coexisting conditions that can be differently accounted. Nonetheless, a different impact on the elderly population, with respect to the pediatric population that shows a mild disease or an asymptomatic infection has been strikingly reported (1, 2). The analysis of SARS-CoV-2 infection incidence and severity indicates, in particular, two intriguing clinical aspects: (1) high mean age of infected patients with higher mortality in patients >65 years old; (2) lowest SARS-CoV-2 incidence in children with rare cases of severe disease (3). This was also observed in SARS-CoV and MERS-CoV infection (4, 5). During SARS-CoV epidemic, the overall mortality was about 15%; however, the stratified analysis by age, showed <1% mortality in younger patients (<24 years old) and a mortality rate above 50% in patients aged above 65 years (6).

By contrast, children have been reported to rarely develop a severe complication such as Kawasaki disease-like vasculitis (7).

## Current Hypotheses Associated With Mortality Rate and Disease Severity

Although the reasons accounting for the differences in disease severity and lethality according to age are not yet established, a few main hypotheses were suggested to explain these differences between children and adult individuals (8, 9), which might explain the limited number of severe cases of COVID-19 in children.

In addition to these tentative explanations, some authors suggested that the low impact of SARS-CoV-2 infection in children might be correlated with the measles/rubella/mumps vaccination (10–14). The triple vaccine containing attenuated strains of measles, mumps, and rubella viruses (MMR) was introduced about 50 years ago, and it is administered in two doses at 12–15 months and 4–6 years of age, respectively, even though a modified schedule could be observed in the different national health systems. Sequence analyses also showed a suggestive homology between an important region of S2 fusion protein of SARS-CoV-2 and the fusion protein F domains in measles and mumps viruses. Furthermore, a sequence similarity between the macrodomains with ADP-ribose-1-phosphatase (ADRP) activity in SARS-CoV-2 non-structural protein 3 (NSP3) and the rubella virus p150 protein was described. These two sequences showed 29% amino acid identity with some functional conserved residues, suggesting that ADRP may play an important role in the rubella virus and SARS-CoV-2 infections. The rubella macrodomain has surface-exposed conserved residues and is included in the attenuated rubella vaccine virus. Interestingly, the anti-rubella antibodies significantly increase in the severe COVID-19 cases with respect to mild COVID-19 cases (15). These amino acid sequence homologies belong to protein regions that could induce an immunological response in the vaccinated individuals against these viruses that may then attenuate the clinical impact of SARS–CoV-2 infection through B- or T-cell responses.

Another clue of the possible role of vaccinations in the comprehension of different clinical impact in patients with SARS-CoV-2 was raised in a study by Miller and coworkers suggesting a link between Bacillus Calmette–Guerin (BCG) vaccination and acquired non-specific immunity against SARS-CoV-2 (16). This study indicates that the countries with specific and long-standing BCG vaccination showed a reduced morbidity and mortality with respect to countries without the BCG vaccination policies.

Although these observations are intriguing and deserve consideration, they would mention only some factors of this jigsaw puzzle and do not fully explain why children appear to be protected against severe SARS-CoV-2 as well as SARS-CoV and MERS-CoV infections, irrespectively of the geographical region and the correlated vaccination policy. In our humble opinion, an additional hypothesis can be raised, certainly not mutually exclusive with the previous ones. Particularly, the sequence similarity between Measles Virus F protein and SARS-CoV-2 S2 protein prompted us to investigate whether a still higher sequence similarity might exist with other human Coronavirus proteins.

The human Coronavirus family is composed by seven viruses: two belonging to alpha coronaviruses (HCoV-NL63 and−229E) and five to beta coronaviruses (HCoV-OC43, HCoV-HKU1, SARS-CoV, SARS-CoV-2, MERS-CoV).

HCoV-NL63, HCoV-229E, HCoV-OC43, and HCoV-HKU1 are associated with mild respiratory disease in immune competent patients with the onset of seasonal epidemics worldwide. These human coronaviruses are considered the second major cause of the common cold surpassed only by Rhinoviruses. In particular, HCoV-OC43 and HCoV-HKU1 induce outbreaks of respiratory diseases especially in temperate regions, and studies in the USA have demonstrated that HCoV-OC43 and HCoV-HKU1 infections are mainly observed at the ages of 1 and 2 years, respectively (17, 18). The infection by respiratory human Coronaviruses is detectable in the first years of human life, and the seroconversion of at least one out of four Coronavirus is detectable in the first two years. Some studies have shown that 50–75% of the population has antibodies against either HCoV-OC43 or HCoV-NL63 in the first two years (19–21). The incidence of infection is higher for HCoV-OC43 and HCoV-NL63 and, interestingly, early infections due to these two viruses protect against subsequent infections against HCoV-HKU1 and HCoV-229E, respectively, whereas the contrary effect was not observed (20). This indicates a complex immunological relationship and cross-protection during infections of similar viruses. In addition, SARS-CoV infection can yield neutralizing antibodies to HCoV-OC43, and vice versa, HCoV-OC43 is able to generate cross-reactive antibodies that recognize SARS-CoV antigens (22, 23).

A study of humoral response against SARS-CoV and MERS-CoV has demonstrated that the neutralizing antibodies rapidly declined, and in the ELISA analysis done 2 years later on the same subjects indicated that the antibody titers were undetectable or close to background levels (17, 24). Similarly, the humoral immunological response just declined 1 year after the infection by HCoV-OC43 and HCoV-HKU1 (20). It is noteworthy that the seasonal HCoV- can infect the adults, and the studies have demonstrated that these viruses induce 22–25% of acute respiratory diseases (21, 25). These data suggest that several HCoV- infections affect the adults who were exposed to viral infection in the first years of life, even though the clinical development is characterized by mild symptoms and also asymptomatic infections take place. Therefore, the respiratory seasonal HCoV- reinfections may be associated with a subprotective or waning of immunological response that is not able to neutralize the infection but may minimize disease aggressiveness.

Overall, the frequent reinfections that occur in children may suggest that they are less susceptible to severe infections due to a cross-response to some respiratory coronavirus antigens. Particularly, beta coronaviruses, such as HCoV-OC43 and HCoV-HKU1, could show larger homologies with SARS-CoV-2. These two viruses, remarkably, are supposed to infect at least 70 to 80% of children, being recognized in around 5% of hospitalized pediatric patients (4).

## Protein Identity Among Human Coronaviruses

SARS-CoV-2 is the seventh human coronavirus reported after HCoV-NL63, HCoV-OC43, HCoV-HKU1, HCoV-229E, SARS-CoV, and MERS-CoV. The first four respiratory coronaviruses cause respiratory manifestations, especially in the upper airways, and do not represent a real clinical problem due to poor symptomatic outcome, even though in some cases, as in immunocompromised patients, they can play an important pathogenic role. However, SARS-CoV and MERS-CoV elicit more aggressive respiratory disorders, characterized by a significantly higher complications rate (e.g., pneumonia) and mortality. However, their spread in the populations has been definitely less wide, especially as far as MERS-CoV is concerned.

Alignment of virus proteins or target fragment of the virus proteins was accomplished using either FASTA (version 36.3.8g, https://faculty.virginia.edu/wrpearson/fasta/fasta36/) or BLAST (version 2.11.0; https://ftp.ncbi.nlm.nih.gov/blast/executables/blast+/LATEST/) softwares. Both FASTA and BLAST programs have the same goal: to identify statistically significant sequence similarity that can be used to infer homology. The homology results rely on two different scores: E-value and bit score. The expected (E) value is a parameter that describes the number of hits one can “expect” to see by chance when searching a database of a particular size. High E values have a higher probability of occurring in the database purely by chance. The bit score is a normalized raw score to the statistical parameters of the scoring system (i.e., length of query and library sequences) and allows for alignment comparisons between independent searches.

It is interesting to note that SARS-CoV and SARS-CoV-2 have a high identity degree of their genomic sequences (with about 90% sequence identity). Since SARS-CoV, SARS-CoV-2, MERS CoV, HCoV-OC43, and HCoV-HKU1 are beta coronaviruses, we aimed to study the amino acid alignment of proteins such as S and N, which are structural proteins with high homology in all coronaviruses, with relevant immunogenic properties (i.e., contain strong epitopes for B and T cells) in SARS-CoV-2 (26). SARS-CoV-2 showed higher identity for N and S aminoacidic sequences with SARS-CoV (90.5 and 76%, respectively), whereas the identity with other beta coronaviruses was around the 30% for N and S, with the exception of N protein that had an identity of 50% with MERS-CoV N protein (Tables 1, 2). It is noteworthy that the analysis of aminoacidic identity of S2 subregion of S protein has demonstrated that the SARS-CoV-2 has strong similarity values with SARS-CoV (90%), MERS-CoV (44.2%), HCoV-OC43 (42.5%), and HCoV-HKU1 (40.3%) (Table 3). On the other hand, the aminoacidic identity on S1 subregion is lower; in fact, the identity with SARS-CoV, MERS-CoV, HCoV-OC43, and HCoV-HKU1 are 66.5, 23.6, 22.5, and 21.7%, respectively. These data suggest that S2 is the subregion more conserved among the different human Coronaviruses, and its importance in the fusion step of viral infection indicates this protein as a valuable target for immune response.

**Table 1:** Identity and similarity score among virus N proteins.

	**SARS-CoV-2**	**SARS-CoV**	**HCoV-HKU1**	**HCoV-OC43**	**HCoV-NL63**	**HCoV-229E**	**MERS-CoV**
SARS-CoV-2							
SARS-CoV	90.5 (97.2)						
HCoV-HKU-1	33.3 (64.1)	35.6 (64.2)					
HCoV-OC43	34.8 (61.7)	37.4 (63.1)	64.2 (85.0)				
HCoV-NL63	34.0 (65.3)	33.0 (64.8)	29.8 (58.9)	33.6 (59.2)			
HCoV-229E	28.5 (57.9)	26.9 (58.0)	31.8 (54.4)	30.4 (57.1)	46.6 (71.6)		
MERS-CoV	50.0 (75.9)	49.9 (75.3)	33.3 (65.3)	38.1 (65.8)	31.1 (59.6)	28.3 (52.7)	

Identity is the amount of identical amino acids between the two proteins; similarity describes the similarity of two protein sequences taking into account the chemical properties of the amino acids and including gaps. Identity and Similarity scores have been estimated using the FASTA program.

Identity % (similarity %).

**Table 2:** Identity and similarity score among virus S proteins.

	**SARS-CoV-2**	**SARS-CoV**	**HCoV-HKU1**	**HCoV-OC43**	**HCoV-NL63**	**HCoV-229E**	**MERS-CoV**
SARS-CoV-2							
SARS-CoV	76.0 (91.5)						
HCoV-HKU1	29.2 (59.0)	29.3 (59.6)					
HCoV-OC43	30.5 (58.2)	30.0 (58.6)	63.3 (84.9)				
HCoV-NL63	30.3 (59.8)	29.3 (59.8)	32.8 (63.2)	28.8 (58.3)			
HCoV-229E	32.2 (63.0)	28.9 (58.0)	32.5 (62.3)	30.8 (58.4)	64.1 (85.8)		
MERS-CoV	31.5 (61.1)	34.1 (63.1)	32.0 (60.8)	31.9 (59.2)	32.3 (59.4)	32.4 (59.6)	

See Table 1 for a definition of identity and similarity scores. Identity % (similarity %).

**Table 3:** Protein S—aminoacidic identity and similarity scores between human coronaviruses and SARS-CoV-2 S protein.

	**SARS-CoV-2 Protein S**	**SARS-CoV-2 S1-region**	**SARS-CoV-2 S2-region**
SARS-CoV	76.0 (91.5)	66.5 (87.7)	90.0 (98.1)
HCoV-HKU1	29.2 (59.0)	21.7 (50.1)	40.3 (73.0)
HCoV-OC43	30.5 (58.2)	22.5 (48.5)	42.5 (72.7)
HCoV-NL63	30.3 (59.8)	25.7(52.0)	33.5 (65.9)
HCoV-229E	32.2 (63.0)	28.2 (56.3)	35.0 (66.5)
MERS-CoV	31.5 (61.1)	23.6 (53.0)	44.2 (74.0)

Scores were calculated on the entire protein length (YP_009724390.1) or on the subregion S1 (P0DTC2 pos: 13-685) or S2 (P0DTC2 pos: 686-1237). Each number indicates identity percentage followed by similarity percentage in parenthesis. Identity and similarity score have been defined in Table 1.

We then sought to analyze the SARS-CoV-2-derived T- and B-cell epitopes of either protein N or S as defined by Grifoni et al. (27). Remarkably, we recognized a statistically significant degree of identity in the human beta coronaviruses and partially in alpha coronaviruses as shown in Tables 4–7.

**Table 4:** Sequence identity of S proteins of human alpha- and beta-coronaviruses against SARS-CoV-2-derived T-cell epitopes (evaluated by blastp program).

	**SARS-CoV-2 S protein epitopes (protein start-end)**	**Identity (%)**	**Mapped start**	**Mapped end**	**E-value**	**BIT-score**
HCoV-HKU1	S_747-763	46.6	829	843	6.97e-04	21.6
	S_747-763	77.7	603	611	0.054	16.2
	S_936-952	52.9	1,021	1,037	0.001	20.8
	S_1101-1115	40.0	1,187	1,201	0.002	20.0
HCoV-OC43	S_747-763	46.6	827	841	7.30e-04	21.6
	S_936-952	47.0	1,020	1,036	0.018	17.3
HCoV-229E	S_304-321	53.8	40	52	0.026	16.9
	S_747-763	41.6	542	553	0.052	16.2
	S_936-952	56.2	822	837	5.64e-04	21.6
	S_996-1004	77.7	881	889	0.006	17.7
	S_1192-1200	55.5	1,094	1,102	0.015	16.5
HCoV-NL63	S_747-763	50.0	799	814	0.004	19.6
	S_936-952	41.1	1,002	1,018	0.009	18.5
	S_996-1004	77.7	1,062	1,070	0.007	17.7
	S_1101-1115	41.6	152	163	0.046	16.2
MERS-CoV	S_304-321	44.4	352	369	4.41e-04	21.9
	S_747-763	43.7	816	831	3.05e-04	22.3
	S_1192-1200	87.5	1,276	1,283	0.008	17.7
	S_1220-1228	75.0	1,303	1,310	0.007	17.7

Homology values are considered significant when showing an E-value <0.05, or since the database is composed of a small number of short fragments (SARS-CoV-2 derived cell epitopes) when showing an arbitrarily bit-score ≥14. The table shows the homologies identified with an E-value <0.10.

**Table 5:** Sequence Identity of N proteins of human alpha- and beta-coronaviruses against SARS-CoV-2-derived T-cell epitopes (evaluated by blastp program).

	**SARS-CoV-2 N protein epitopes (protein start-end)**	**Identity (%)**	**Mapped start**	**Mapped end**	**E-value**	**BIT-score**
HCoV-HKU1	N_138-146	80.0	421	425	0.046	14.6
HCoV-OC43	N_138-146	66.6	155	160	0.093	13.9
HCoV-229E	N_138-146	57.1	325	331	0.087	13.9
	N_265-274	83.3	260	265	0.044	14.6
HCoV-NL63	-	-	-	-	-	-
MERS-CoV	N_265-274	60.0	257	266	0.002	18.1

Homology values are considered significant when showing an E-value <0.05, or since the database is composed of a small number of short fragments (SARS-CoV-2 derived cell epitopes) when showing an arbitrarily bit-score ≥14. The table shows the homologies identified with an E-value <0.10.

**Table 6:** Sequence identity of S protein of human alpha- and beta-coronaviruses against SARS-CoV-2-derived B-cell epitopes (evaluated by blastp).

	**SARS-CoV-2 S protein epitopes (protein start-end)**	**Identity (%)**	**Mapped start**	**Mapped end**	**E-value**	**BIT-score**
HCoV-HKU1	S_287-317	40.0	278	307	2.81e-05	26.6
	S_524-598	25.3	611	681	1.61e-05	29.3
	S_888-909	50.0	977	994	3.62e-05	25.4
HCoV-OC43	S_287-317	42.8	286	313	4.84e-06	28.5
	S_524-598	28.1	611	681	1.09e-06	32.7
	S_888-909	44.4	976	993	1.69e-04	23.5
HCoV-229E	S_524-598	27.6	434	497	9.22e-05	26.9
	S_888-909	36.8	760	778	0.002	20.8
HCoV-NL63	S_524-598	36.1	613	648	4.99e-06	30.8
	S_802-819	50.0	1,224	1,235	0.054	16.2
	S_888-909	36.8	941	959	0.004	19.6
MERS-CoV	S_287-317	43.3	336	365	3.27e-08	34.7
	S_524-598	29.0	603	633	0.048	19.6
	S_888-909	42.8	963	983	1.56e-04	23.9

Homology values are considered significant when showing an E-value <0.05, or since the database is composed of a small number of short fragments (SARS-CoV-2 derived cell epitopes) when showing an arbitrarily bit-score ≥16. The table shows the homologies identified with an E-value <0.10.

**Table 7:** Sequence identity of N protein of human alpha- and beta-coronaviruses against SARS-CoV-2-derived B-cell epitopes (evaluated by blastp).

	**SARS-CoV-2 N protein epitopes (protein start-end)**	**Identity (%)**	**Mapped start**	**Mapped end**	**E-value**	**BIT-score**
HCoV-HKU1	N_42-62	45.0	54	73	0.003	18.9
	N_153-172	52.9	170	186	5.61e-05	23.5
	N_153-172	71.4	54	60	0.013	16.9
HCoV-OC43	N_42-62	57.1	64	77	0.003	18.5
	N_153-172	45.0	168	187	2.83e-05	24.3
HCoV-229E	-	-	-	-	-	-
HCoV-NL63	-	-	-	-	-	-
MERS-CoV	N_42-62	70.0	33	52	2.31e-09	35.8
	N_153-172	55.0	142	161	6.16e-06	26.2
	N_355-401	34.1	353	393	0.004	20.0

Homology values are considered significant when showing an E-value <0.05, or since the database is composed of a small number of short fragments (SARS-CoV-2-derived cell epitopes) when showing an arbitrarily bit-score ≥14. The table shows the homologies identified with an E-value <0.10.

This homology could be consistent with the formation of some antibodies directed against respiratory coronaviruses, which can protect, albeit partially, against SARS-COV-2. Since Coronavirus infection usually occurs in the early years of life and the immune response from Coronavirus is not maintained for a long time, this could explain the resistance of children and not in the elderly. In this light, based on genetic identities across coronaviruses, the establishment of specific immunity against one of the four common strains might partially protect against from the other ones, including SARS-CoV, MERS-CoV, and SARS-CoV-2. This would easily explain why only few children are symptomatic, why the disease course is overall milder in this category, and why asymptomatic carriers have been frequently observed among children (28–31).

In addition, we have seen how conserved sequences can be present in putative epitopes for B- and T-cells (27). The same procedure was used for the N protein, as shown in Table 4. Several putative epitopes of SARS-CoV-2 are conserved in HCoV-OC43 and HCoV-HKU1, thus reinforcing the possibility of cross-reaction. Interestingly, some reports indicated a correlation between HCoV-OC43 and SARS-CoV-2 for T-antigens (27, 32, 33). These observations sustain the hypothesis that early infections by beta coronaviruses may induce a cross-reactive immune response and influence the development of disease using both B- and T-cell response.

Of note, we have aligned (Table 8) SARS-CoV-2 S1 and S2 amino acid sequences with measles virus F1 protein, as recently emphasized, mumps virus F1 protein, and rubella virus E1 protein. In all cases, we demonstrated a lower identity than for human respiratory coronaviruses was observed (Table 8 and Supplementary Table 1).

**Table 8:** Protein S—aminoacidic identity and similarity between SARS-CoV-2 S protein (YP_009724390.1) and its subregions S1 (**P0DTC2 13-685**) and S2 (**P0DTC2 686-1237**) [identity % (similarity %)] with measles virus F1 protein, mumps virus F1 protein, and rubella virus E1 protein.

	**SARS-CoV-2** **Protein S**	**SARS-CoV-2** **S1-region**	**SARS-CoV-2** **S2-region**
Measles Virus F protein NP_AGA17217	22.5 (51.3)	20.7 (56.0)	22.5 (51.3)
Mumps Virus F1 protein NP_054711.1	17.3 (50.8)	25.0 (65.6)	17.3 (50.8)
Rubella Virus E1 protein NP_740664.1	22.5 (50.0)	24.3 (51.4)	22.5 (50.0)

## Discussion

The set of these observations leads us to define how it is possible that infections by coronaviruses, such as HCoV-OC43, could lead to an immune response that allows or, more precisely, contributes to the child having pathology in the worst mild scenario. The respiratory Coronavirus infection and the vaccination against rubella/mumps/measles could put children in optimal conditions for a prompter immunological defense against SARS-CoV-2. In particular, IgA might play a significant protective role as shown in SARS-CoV and MERS-CoV infections (34, 35).

When compared to its SARS-CoV and MERS-CoV, SARS-CoV-2 seems to have greater infectivity (higher R_0_) but less lethality. It has many similarities to the SARS-CoV virus, which in 2003 killed 774 people and infected more than 8,000, with very similar symptoms: fever, cough, headache, breathing difficulties, and pneumonia. Even for SARS-CoV, there were few cases among children: only 80 cases confirmed in the laboratory and 55 probable or suspect cases. In a 2007 report, it is documented that children under 12 had milder SARS-CoV-related symptoms than adults. Relatively few children or teenagers died from this Coronavirus. Similarly, during the MERS outbreak in 2016, it was reported that the virus was rare in children, although the “reason for the low prevalence was not known (36).”

## Conclusion

In conclusion, we hypothesize that previous infections from other beta coronaviruses may confer partial protection from SARS-CoV-2 through some degrees of cross-immunity, especially in pediatric patients. Hence, the onset of cross-reactivity among the different beta coronaviruses may play an important role in the attenuation of disease severity and then clinical impact on children. This cross-immunity, comprising both T- and B-cell compartments, may be possibly increased by vaccination with measles/mumps/rubella vaccination through an important homology of some rubella and paramyxovirus proteins with respect to SARS-CoV-2 S protein. To fully understand the complex mechanisms underlying the relative resistance to SARS-CoV-2 observed in children, it is now warranted to perform functional as well as epidemiological studies incorporating serology for the commonest beta coronaviruses.

## Data Availability Statement

The original contributions presented in the study are included in the article/Supplementary Material, further inquiries can be directed to the corresponding author.

## Author Contributions

PP and DG conceptualized and designed the study and wrote the manuscript. GM and MN performed the genetic analysis. ED analyzed the data. EC critically revised the manuscript. All authors contributed to the article and approved the submitted version.

## Conflict of Interest

The authors declare that the research was conducted in the absence of any commercial or financial relationships that could be construed as a potential conflict of interest.
