# Supplementary material for: Cross-Immunization Against Respiratory Coronaviruses May Protect Children From SARS-CoV2: More Than a Simple Hypothesis?
Source: Front Pediatr. 2021 Jan 18;8:595539. doi: 10.3389/fped.2020.595539 (PMC7849449; doi:10.3389/fped.2020.595539)
Supplement: Supplementary file 1 [file Table_1.docx]

**Supplementary Table 1.** Alignment of SARS- CoV-2 S2 region with measles and mumps F1 protein, rubella virus E1 protein, SARS-CoV S2 region and HCoV-OC43 S2 protein.

gi|25121516|ref|NP_740664.1| --------------------------------------------------EEAFTYLCT- 9

YP_009555241.1:780-1350 ------------SL----EPVGGLYEIQIPSEFTIGNMVEFIQTSSPKVTIDCAAFVCGD 44

sp|P59594|668-1255 STSQKSIVAYTMSLGADSSIAYSNNTIAIPTNFSISITTEVMPVSMAKTSVDCNMYICGD 60

gi|429845388|gb|AGA17217.1| ----------------------------------MGLKV--------------------- 5

gi|9695420|ref|NP_054711.1| ------------------------------------------------------------ 0

gi|25121516|ref|NP_740664.1| ------------------------------------------------------------ 9

YP_009555241.1:780-1350 YAACKSQLVEYGSFCDNINAILTEVNELLDTTQLQVANSLMNGVTLSTKLKDGVNFNVDD 104

sp|P59594|668-1255 STECANLLLQYGSFCTQLNRALSGIAAEQDRNTREVFAQVKQ-MYKTPTLKY-----FGG 114

gi|429845388|gb|AGA17217.1| -------------------------------NISAVF--------MAVLLTLQ--TPAGQ 24

gi|9695420|ref|NP_054711.1| ---------------------------------MKVF--------LVTCLGFAVFSSSVC 19

gi|25121516|ref|NP_740664.1| -------APGCATQAPVPVRLAGVR----FE------SKIVDGGCFAPWDLEATGACICE 52

YP_009555241.1:780-1350 --INFSPVLGCLG--SECSKA---SSRSAIEDLLFDKVKLSDVGFVEAY-----NN---- 148

sp|P59594|668-1255 --FNFSQIL------PDPLKP---TKRSFIEDLLFNKVTLADAGFMKQY-----GE---- 154

gi|429845388|gb|AGA17217.1| IHWGNLSKIGVVGIGSASYKVMTRSSHQSLVIKLMPNITLL-------------NN---- 67

gi|9695420|ref|NP_054711.1| VNINILQQIGYIKQQVRQLSYYSQSSSSYIVVKLLPNIQPTD------------NS---- 63

: .

gi|25121516|ref|NP_740664.1| IPTDVSCEGLGAWVPAAPCARIWNGTQRACTFWAVNAYSSGGYAQLASYFNPGGSYYKQY 112

YP_009555241.1:780-1350 ------CTGGA-EIRDLICVQSYKGI--KVLPPLLSENQISGYTL--------------- 184

sp|P59594|668-1255 ------CLGDI-NARDLICAQKFNGL--TVLPPLLTDDMIAAYTA--------------- 190

gi|429845388|gb|AGA17217.1| ------CTRV--------EIAEYRRLLRTVLEPIRD--ALNAMTQ-------------NI 98

gi|9695420|ref|NP_054711.1| ------CEFK--------SVTQYNKTLSNLLLPIAE--NINNIA---------------- 91

* :. :

gi|25121516|ref|NP_740664.1| HPTACEVEPAFGHSDAACWGFPTDTVMSVFALASYVQHPH-----------------KTV 155

YP_009555241.1:780-1350 ----AATSA----SLFPPWTAAAG----V---PFYLNVQYRINGLGVTMDV----LSQNQ 225

sp|P59594|668-1255 ----ALVSG----TATAGWTFGAGAALQI---PFAMQMAYRFNGIGVTQNV----LYENQ 235

gi|429845388|gb|AGA17217.1| RPV-QSVASSRRHKRFAGVV-LAGAALGV---ATAAQITA---GIALHRSMLNSQAIDNL 150

gi|9695420|ref|NP_054711.1| ----SPSSGSRRHKRFAGIA-IGIAALGV---ATAAQVTA---AVSLVQAQTNARAIAAM 140

. : :

gi|25121516|ref|NP_740664.1| RVKFHTETRTVWQLS---------VAGVSCNVTTEHPFCNTPHGQLEVQVPPDPGDLVEY 206

YP_009555241.1:780-1350 KLIANAFNNALYAIQEGFDATNSALVKIQAVVNANA---E-ALNNLLQQL---------- 271

sp|P59594|668-1255 KQIANQFNKAISQIQESLTTTSTALGKLQDVVNQNA---Q-ALNTLVKQL---------- 281

gi|429845388|gb|AGA17217.1| RASLETTNQAIEAIRQAGQEMILAVQGVQDYINNEL---IPSMNQLSCDL---------- 197

gi|9695420|ref|NP_054711.1| KNSIQATNRAVFEVKEGTQRLAIAVQAIQDHINTIM---NTQLNNMSCQI---------- 187

: . ..:: : : :. :. . : ::

gi|25121516|ref|NP_740664.1| IMNYTGNQQSRWGLGSPNCHGPDWASPVCQRHSPDC-SRLVGAT---------------- 249

YP_009555241.1:780-1350 ----------------SNRFG---------------------AI-SASLQEILSRLDALE 293

sp|P59594|668-1255 ----------------SSNFG---------------------AI-SSVLNDILSRLDKVE 303

gi|429845388|gb|AGA17217.1| IGQKLGLKLLRYYTEILSLFGPSLRDPISAEISIQALSYALGGDINKVLEKLGY-SGGDL 256

gi|9695420|ref|NP_054711.1| LDNQLATSLGLYLTELTTVFQPQLINPALSPISIQALRSLLGSMTPAVVQATLS-TSISA 246

. . .

gi|25121516|ref|NP_740664.1| PE-------RPRLRLVDA---------DDPLLRTAPGPG---EVWVTPVIGSQARKCGLH 290

YP_009555241.1:780-1350 AEAQIDRLINGRLTALNAYVSQQLSDST---LVKFS--AAQAMEKVNECVKSQSSRIN-- 346

sp|P59594|668-1255 AEVQIDRLITGRLQSLQTYVTQQLIRAA---EIRAS--ANLAATKMSECVLGQSKRVD-- 356

gi|429845388|gb|AGA17217.1| LGILESRGIKARITHVDTESYFIVLSIAYPTLSEIKGVIVHRLEGVSYNIGSQEWYTTVP 316

gi|9695420|ref|NP_054711.1| AEILSAGLMEGQIVSVLLDEMQMIVKINIPTIVTQSNALVIDFYSISSFINNQESIIQLP 306

:: : :. : .*

gi|25121516|ref|NP_740664.1| IRAGPYGHATVEMPEWIHAHTTSDPWHPPGPLGLKFKT---------------------- 328

YP_009555241.1:780-1350 --FCGNGNHIISLVQ------N----APYGLYFIHFSYVPTKYVTAR--VSPGLCIAGDR 392

sp|P59594|668-1255 --FCGKGYHLMSFPQ------A----APHGVVFLHVTYVPSQERNFT--TAPAICHEGK- 401

gi|429845388|gb|AGA17217.1| KYVATQGYLISNFDE------SSCTFMPEGTV-------CSQNALYPMSPLLQECLRGST 363

gi|9695420|ref|NP_054711.1| DRILEIGNEQWSYPA------KNCKLTRHHIF-------CQYNEAERLSLESKLCLAGNI 353

* .

gi|25121516|ref|NP_740664.1| -------------------VRPVALPRTLAPPRNVRVTGCYQ----CGTPA--LVEGLAP 363

YP_009555241.1:780-1350 GIAPKSGYFVNVNNTWMYTGSGYYYPEPITENNVVVMSTCAVNYTK---APYVMLNTSIP 449

sp|P59594|668-1255 AYFPREGVFVFNGTSWFITQRNFFSPQIITTDNTFVSGNCDVVIGI---INNTVYDPLQP 458

gi|429845388|gb|AGA17217.1| KSCART------------------------------------------------------ 369

gi|9695420|ref|NP_054711.1| SACVFS----PIAGS--------YMRRFVALDGTI-VANCRSLTCLCKSPSYPIYQPDHH 400

gi|25121516|ref|NP_740664.1| GGGN-----C-HLTVNGEDLGAVPPGKF-----VTAALLNTPPPYQVSCGGESDRATARV 412

YP_009555241.1:780-1350 NLPDFKEELDQW--FKNQTSVAPDLS-L-----------DYINVTFLDLQVEMNRL---- 491

sp|P59594|668-1255 ELDSFKEELDKY--FKNHTSPDVDLGDI-----------SGINASVVNIQKEIDRL---- 501

gi|429845388|gb|AGA17217.1| ------------------------------------------------------------ 369

gi|9695420|ref|NP_054711.1| AVTTIDLTACQTLSLDGLDFSIVSLSNITYAENLTISLSQTINTQPIDISTELSKVNASL 460

gi|25121516|ref|NP_740664.1| IDPAAQSFTGVVYGTHTTAVSETRQTWAEWAAAHWWQLTLGAICALPLAGLLACCAKCLY 472

YP_009555241.1:780-1350 Q-EAI---KVLNQSYINLKDIGTYEYYVKWPWYVWLLICLAGVAMLVLLFFICCCTGCGT 547

sp|P59594|668-1255 N-EVA---KNLNESLIDLQELGKYEQYIKWPWYVWLGFIAGLIAIVMVTILLCCMTSCCS 557

gi|429845388|gb|AGA17217.1| ------------------------------------------------------------ 369

gi|9695420|ref|NP_054711.1| Q-NAV---KYIKESNHQLQSVNVNS---KIGAIIVAALVLSI-LSIIISLLFCCWAYVAT 512

gi|25121516|ref|NP_740664.1| -YLRGAIA-------------PR----------- 481

YP_009555241.1:780-1350 SCFK---KCGGCCDDYTGYQELVIKT--S----- 571

sp|P59594|668-1255 -CLKGACSCGSCCKFDEDDSEPVLKG--VKLHYT 588

gi|429845388|gb|AGA17217.1| ---------------------------------- 369

gi|9695420|ref|NP_054711.1| KEIRRI-------NFKTNHINTISSSVDDLIRY- 538

gi|25121516|ref|NP_740664.1| Rubella

YP_009555241.1:780-1350 OC43 (S2)

sp|P59594|668-1255 S2 - CoV2

gi|429845388|gb|AGA17217.1| Measles

gi|9695420|ref|NP_054711.1| Mumps
